# Supplementary material for: Individualized prediction of COVID-19 adverse outcomes with MLHO
Source: Sci Rep. 2021 Mar 5;11:5322. doi: 10.1038/s41598-021-84781-x (PMC7935934; doi:10.1038/s41598-021-84781-x)
Supplement: Supplementary file 1 — Supplementary Information. [file 41598_2021_84781_MOESM1_ESM.docx]

Individualized Prediction of COVID-19 Adverse Outcomes with MLHO

Hossein Estiri, Zachary H. Strasser, Shawn N. Murphy

**Table 1S.** Demographics of the COVID-19 patient population at MGB.

|  | **Count** | **Hospitalization** | **ICU** | **Ventilation** | **Mortality** |
| --- | --- | --- | --- | --- | --- |
| **Overall** | >13,300 | 26% | 10% | 4.5% | 5.3% |
| **Black or African American** | >1,900 (~15%) | 29% | 11% | 5.5% | 4.5% |
| **Hispanic** | >1,300  (~10%) | 15.9% | 4.9% | 3% | 1.8% |
| **White** | >7,300 (~54%) | 27.5% | 12.9% | 4.5% | 7.3% |
| **Female** | >7,800 (~58%) | 22.8% | 8.7% | 3 % | 4.3% |
| **Male** | >5,600 (~41%) | 29.6% | 13.6% | 6.5% | 6.7% |
| **Age** | 50.6*  (21.2**) | 62.8*  (19.1**) | 64.9*  (16.7**) | 64.3* (14.4**) | 78*  (14**) |

* mean, years

** standard deviation, years

**APPENDIX**

**Testing for potential prediction bias**

In our experience using clinical data for modeling, we have sometimes encountered biases where predictive algorithms provide more reliable predictions for patients with more clinical records. Such patients often have multiple chronic conditions and hence visit the healthcare systems more frequently, which results in a larger number of data points, compared with the healthier patients. To evaluate such potential biases in our predictive algorithms, we calculated the squared prediction error – actual status (0 or 1) – predicted probability of the outcome – in each of models and computed the mean squared error (MSE) for each patient over the 10 model iterations. We then plotted the MSE against the total number of clinical records and fitted a smoothed line to identify any potential trend (bias) – Figure S1.


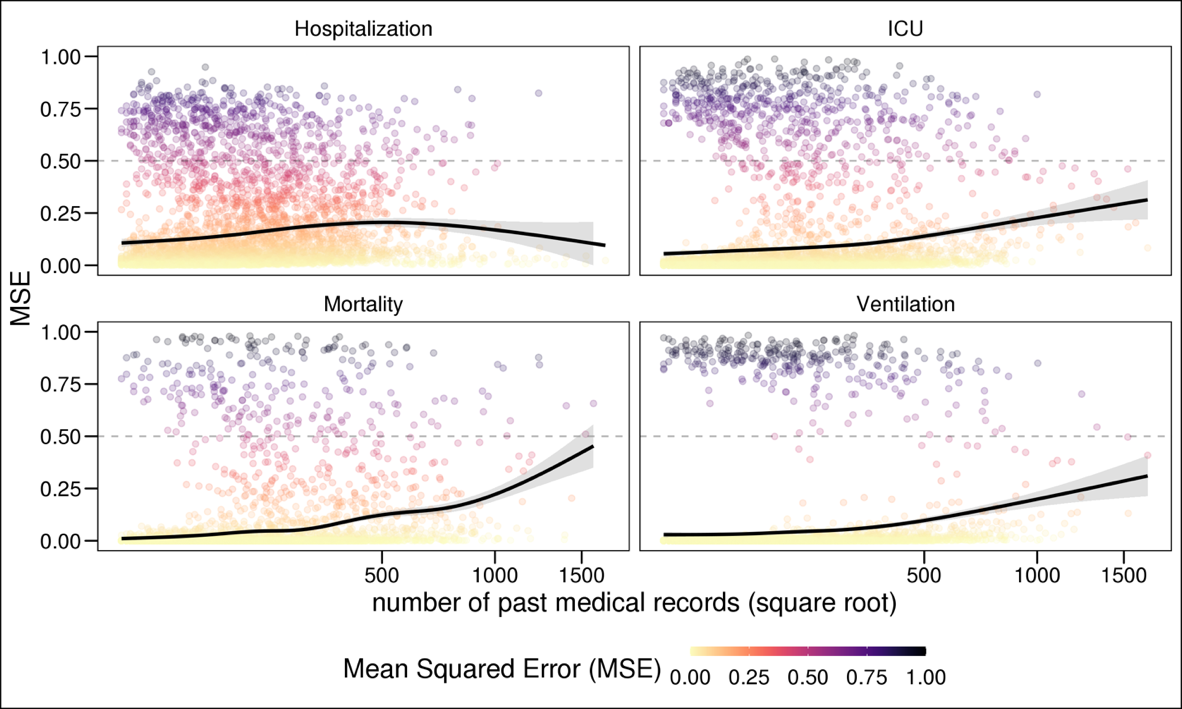


**Figure S1.** Evaluating the prediction bias
